# Isolation and Control of Fruit and Vegetable Rot Fungi

**DOI:** 10.3390/jof10080539

**Published:** 2024-08-01

**Authors:** Xiaoli Tan, Nengguo Tao

**Affiliations:** School of Chemical Engineering, Xiangtan University, Xiangtan 411105, China

Fruits and vegetables play an important role in people’s dietary health and economic development. However, fresh fruits and vegetables, due to their richness in nutrients, high water content, and active metabolism, are susceptible to pathogens at multiple stages including the planting, transportation, storage, and marketing processes, resulting in severe postharvest product decay and huge economic losses. In addition, there is a wide variety of pathogenic fungi, and the postharvest pathogenic species of fruits and vegetables vary depending on several factors, such as the type of fruit or vegetable, the variety, the geographic region, and the time of storage [1,2]. Therefore, isolation of the pathogens from specific postharvest products is essential for subsequent investigation of their pathogenic mechanisms and the development of strain-specific preservation measures. Wang et al. isolated *Diaporthe passiflorae* from rotting passion fruit brought from Fujian (China) and found that it could cause yellowing and increased cell membrane permeability at the infested site of postharvest passion fruit, leading to severe fruit decay [3]. Lv et al. isolated nine fungi causing postharvest disease in fresh *Codonopsis pilosula* and found that *Mucor* was first observed on day 7, followed by root rot caused by *Fusarium* on day 14. Blue mold disease caused by *Penicillum expansum* was detected as the most serious postharvest disease on day 28. Pink rot disease caused by *Trichothecium roseum* was observed on day 56 [1].

The development of highly targeted control strategies can be aided by research into the pathogenic processes of pathogens and the role of key genes. *Penicillium expansum* is a major apple rot pathogenic fungus, and the PacC-pH signaling pathway strongly influences the development, pathogenicity, and malate production of *P. expansum* [4]. Zhuo et al. discovered that three Ena family genes (*PeEnaA*, *PeEnaB*, and *PeEnaC*) are significant downstream targets of *PePacC* [5]. Among them, the PeEna protein family is essential for *P. expansum*’s malate production. Ergosterol is an important component of the cell membrane of filamentous fungi; Han et al. discovered that *P. expansum*’s ergosterol synthesis and spore formation were mediated by all three of the *erg4* genes (*erg4A*, *erg4B*, and *erg4C*) [6]. Furthermore, *erg4B* and *erg4C* were revealed to have a role in spore morphogenesis, cell wall integrity, and the oxidative stress response. To repel infection, the host launches its own defense mechanisms in response to the pathogen’s attack [7,8]. By using proteome sequencing, Xu et al. discovered that *P. expansum* infection boosted the activity and expression of defense enzymes and triggered defensive systems such as apple phenylpropanes, flavonoids, and hormones. When the pathogen attacks the host, the host also activates its own defense mechanisms to resist pathogen infestation [9]. These findings point to the possibility of controlling fruit diseases by enhancing host resistance by fortifying the fruit resistance system, while also preventing the growth and development of pathogens by undermining the function of the pathogens’ essential genes.

Presently, the main control practice for postharvest fruit and vegetable disease is using synthetic chemical fungicides. But the issues surrounding the usage of these chemicals—health, the environment, and the rise of resistant strains—are coming to light more and more. Recent study has shown that biocontrol control agents (BCA), plant essential oils (EOs), and metal ion compounds with safe, effective, and eco-friendly qualities are becoming more and more popular [10,11,12]. The genus *Phytophthora* is destructive to crops, and there are relatively few strategies available to prevent it. Santos et al. have shown that *Trichoderma aggressivum* f. *europaeum*, *T. longibrachiatum*, *Paecilomyces variotii*, and *T. saturnisporum* had highly effective antagonistic activity against *Phytophthora capsici* and *P. parasitica*, which considerably reduced the severity of Phytophthora blight in pepper [12]. Similarly, *Meyerozyma caribbica* can act as a bio-antagonist to impede *Alternaria alternata* growth and reduce the occurrence of jujube black spot rot [13]. BCAs primarily function by either directly or indirectly inhibiting the growth of pathogens; hence, encouraging BCAs’ growth will increase the effectiveness of their control. According to Fan et al. the cell-free supernatant of *Bacillus subtilis* demonstrated remarkable efficacy in stifling the proliferation of *Botryosphaeria dothidea* and reducing its pathogenicity on kiwifruit. Additional research verified that the pathogen is under oxidative stress, which mitigates the severity of kiwifruit soft rot [14]. The production of biofilms has been demonstrated in studies to aid microorganisms in absorbing nutrients more quickly and increasing their competitiveness. By adding CaCl_2_ (5.14 g/L) to the medium, Zheng et al. observed a significant increase in the biocontrol strain *B. mojavensis* D50’s ability to form biofilm, colonize roots, and exhibit antifungal activity, as well as a reduction in the incidence of tomato gray mold [15]. Chen et al. demonstrated that 0.1% β-glucan improved the biofilm-forming ability of marine yeast *Scheffersomyeces spartinae* W9, enhanced its tolerance to various stress, and improved the biocontrol ability of W9 against strawberry gray mold [16]. Additionally, phenylalanine treatment increased the secretion of *M. caribbica* phenylethanol, which in turn encouraged the formation of biofilms, decreased *A. alternata* colonization on jujube fruit, and enhanced the biocontrol efficiency of the fruit against jujube black spot rot [13]. EOs have also been widely used in the control of postharvest diseases of fruits and vegetables. Chen et al. found that perillaldehyde fumigation reduces postharvest rot of sweet potatoes by stimulating the production of excessive ROS as well as inducing severe oxidative damage in *F. solani* [16]. According to Ouyang et al., trans-2-Hexenal can be utilized to suppress *Geotrichum citri-aurantii* and lower the occurrence of citrus sour rot. The primary explanation for trans-2-Hexenal’s antifungal activity could be cell membrane damage brought on by decreased lipid and ergosterol levels [10]. Metal ion compounds such as ferric chloride can be used to control citrus anthracnose by triggering autophagy in *Colletotrichum gloeosporioides* to inhibit its spore germination and reduce pathogenicity [17]. Finding and developing more green control measures for fruit and vegetable diseases and elucidating their mechanisms of action will help to reduce postharvest losses and extend the shelf life of fruits and vegetables.

In conclusion, “Isolation and Control of Fruit and Vegetable Rot Fungi” comprises the latest research findings on the isolation and characterization of fruit and vegetable decay fungi, as well as control strategies. The studies cover a wide range of aspects from biofilm formation, mechanisms of action of antifungal agents, biological characterization of specific pathogens, to the development of biological control strategies. These studies not only provide insights for understanding pathogen–host interactions, but also provide a scientific basis for developing new, environmentally friendly approaches to disease management. Through these research results, it can be expected that the reliance on chemical fungicides will be reduced in the future and sustainable agriculture will be promoted.
